# Exploring Mothers’ Experience of a Linguistic Feedback Technology for Children at Risk of Poor Language Development: Qualitative Pilot Study

**DOI:** 10.2196/27049

**Published:** 2021-08-31

**Authors:** Lydia So, Erin Miller, John Eastwood

**Affiliations:** 1 Department of Community Paediatrics South Western Sydney Local Health District Liverpool Australia; 2 Sydney Local Health District Sydney Australia

**Keywords:** language development, technology, feedback, socioeconomic factors

## Abstract

**Background:**

The early language environment is important for language development and a child’s life-course trajectory. Risk factors associated with poor language development outcomes in children include maternal anxiety and depression, low educational attainment, substance misuse, and low socioeconomic status. Language Environment Analysis (LENA) is a wearable technology designed to promote caregivers’ engagement in supporting their children’s language development. LENA provides quantitative linguistic feedback, which has been shown to improve caregiver language output, thus enhancing a child’s language environment. There is limited research on the uptake of this technology by families with developmentally at-risk children.

**Objective:**

This qualitative study aims to explore the conditions under which mothers with children at risk of poor developmental outcomes are willing to adopt the use of LENA to monitor and improve caregiver language output.

**Methods:**

Using a qualitative interpretive design, semistructured, in-depth interviews were conducted with 8 mothers. Participants were recruited purposively to select the maximal variation of socioeconomic and ethnodemographic backgrounds. The transcribed interview data were analyzed thematically and interpretatively. Themes were mapped abductively to an extended Unified Theory of Acceptance and Use of Technology, which included contextual factors for LENA acceptance.

**Results:**

Factors that influenced the intention to use LENA included both technology-specific acceptance factors and contextual factors. Technology acceptance themes included reassurance, feeling overwhelmed, and trust. These themes were mapped to performance expectancy, effort expectancy, and social influence. Contextual themes included emergent success and the intrusion of past difficulties. These were mapped to parenting self-efficacy and perceived risk. The theme of building on success described behavioral intention. Mothers were more likely to adopt LENA when the technology was viewed as acceptable, and this was influenced by parenting self-efficacy and perceived risk.

**Conclusions:**

LENA is a technology that is acceptable to mothers with children who are at risk of poor language development outcomes. Further studies are needed to establish LENA’s effectiveness as an adjunct to strategies to enrich a child’s early language environment.

## Introduction

### Background

Given the importance of language skills at school entry, successful language development in early childhood is foundational. Parental engagement in language activities has been shown to improve language development and optimize school readiness [1]. The communication environment is reported to be a more dominant predictor of early language than social background [2]. Hoff [3] found that the effect of socioeconomic status (SES) on language development was fully mediated by maternal speech: “[The] critical role of early home language environment as a root cause of academic achievement disparity between children from low and high socioeconomic status homes is undeniable.” A rich language environment is considered foundational to early language development and for future academic performance [2,4,5].

Local research has shown that 1 in 5 Australian children start school behind and are not ready to take advantage of learning opportunities at school [6]. There is a snowball effect, with children beginning school with delayed language being at greater risk of school failure [7]. Early language is an important precursor for development and is linked to literacy, cognitive and educational outcomes, and economic opportunities [7,8]. In particular, early interventions aimed at enriching home language environments and targeting early childhood development are argued to reduce social and health inequities across the life course [9,10] and ameliorate cumulative disadvantage [11].

Multiple factors have been implicated in language development. In particular, barriers to parent-child interaction negatively affect child language development [12]. These risk factors include social disadvantage, parental mental health, and maternal drug use [7,13-18].

The correlation between low SES (as defined by income level and level of education) and poor outcomes has been linked to the quantity and quality of language that a child is exposed to in their home environment [11,19,20]. SES is also a predictor of children’s school readiness and future academic achievement [3,21,22]. Mothers with low SES backgrounds are more likely to use more directives, use fewer open-ended questions, speak in less complex sentences, and produce less speech and gesture. Children from low SES homes often start kindergarten with lower language and literacy skills than those from high SES homes [23,24]. This disparity has been shown to persist throughout school years and is predictive of lower high school graduation rates and economic opportunities [25].

Parental mental health affects the interaction between children and their caregivers. Maternal depression has a negative influence on children’s language development [26,27]. A mother’s engagement in their child’s learning can be undermined by maternal depression, as mothers experiencing more stress and depression speak less to their children [13,28]. Depressed mothers are less sensitively attuned to their children and tend to be less emotionally responsive and less contingent [12,29]. Maternal depression and anxiety are independently associated with language delay [15]. Maternal psychological distress during the perinatal period is negatively associated with language outcomes [10]. Paternal depression has been linked to expressive vocabulary development through reduced parent-to-child reading [30].

Children who are exposed to continuing parental drug use are at risk of developmental delay [17]. Drug abuse is characterized as a chronic relapsing disease [31]. Maternal drug use affects mother-child behavior and the ability to provide a consistent and nurturing environment [32]. A review of studies has shown that prenatal exposure to cocaine is associated with language deficits [18].

A rich language environment, particularly through child-directed speech and adult-child conversations, is positively associated with language development. Infants with more child-directed speech show faster language processing speed and larger vocabularies [33,34]. Conversational turns between children and adults are essential for language development [35].

One approach to supporting early language is through a parent intervention targeted at enriching the home language environment by increasing child-directed speech and contingent responses [14]. Language interventions are often directed at parents as the primary contributors to their children’s language environment. A meta-analysis of 18 studies showed that parent-implemented language interventions can improve language skills in children aged between 18 and 60 months [36]. The most common focus of these interventions was directed at “what the parents say and how much the parents say” [36]. However, adults are generally rarely conscious of the quantity of talk provided to children and frequently overestimate the amount they talk with their children [37]. Studies have demonstrated that caregiver language output can be increased by providing feedback on the linguistic environment, thus enhancing the child’s language environment [37,38]. Therefore, having a tool to measure the linguistic environment and providing feedback over time may complement and enhance parent-implemented language interventions and support caregivers in improving the home language environment.

### Language Environment Analysis

Language Environment Analysis (LENA) provides quantitative feedback on the home language environment and has been likened to a “linguistic pedometer” [37]. LENA is a wearable, digital recording device and software package. The small recording device is worn in the front pocket of specially designed clothing. The recordings are then processed by the LENA software to generate frequency counts of the number of words a child is exposed to, the number of vocalizations the child produces, and the number of conversational turns the child takes with an adult for up to 16 hours. LENA also produces data about the environment, including electronic media, distant speech, background noise, and silence. The LENA-generated feedback can be used to identify patterns in communication that can be discussed during feedback sessions. Total daily counts can be used to monitor progress and set goals much like a pedometer encourages the user to increase their step count. With the clinician, LENA feedback can be used as a basis for discussing ways to improve the language environment. As the software algorithm identifies speech sounds, LENA can be used with non–English-speaking families. LENA has been validated in several languages, including Mandarin, Swedish, Spanish, and French [33,39-41]. Like a pedometer, LENA assists parents to see the language environment they provide for their child, understand their role in this, and reinforce positive behavioral change.

LENA has been shown to influence adult language behavior and thus improve a child’s home language environment. Intervention, including weekly feedback with LENA results, showed a significant and prolonged increase in caregiver language output [37]. This leads to improvements in a child’s language environment. A randomized controlled pilot study for families with low SES using LENA in combination with an intervention curriculum showed significant but short-term increases in both parent language interactions with their children and child language outcomes. These outcomes included increases in daily adult word count, conversational turn count, and number of child vocalizations [42]. Internationally, other parent-led language intervention programs based on LENA in the United States include the Thirty Million Words Initiative [43] and Providence Talks [44] and Talking Matters in New Zealand [45].

### Unified Theory of Acceptance and Use of Technology

Successful implementation of LENA technology is determined by its acceptability to parents with children who are developmentally at risk. Unified theory of acceptance and use of technology (UTAUT) is a simplified theory that explains user acceptance and the use of technology. In this model, technology acceptance predicts its use [46]. Behavioral intention or acceptability is predicted by three antecedents: (1) performance expectancy, which is “the degree to which an individual believes that the system helps improve job performance,” (2) effort expectancy, which is the system’s ease of use, and (3) social influence, which is the “degree to which an individual perceives that important others believe he or she should use the new system” [47]. Various extensions have been made to UTAUT, including the addition of contextual factors [48]. Important contextual factors specific to LENA use by mothers with children who are at risk of poor language development outcomes include perceived risk and parenting self-efficacy. Perceived risk with technology increasingly includes concerns about privacy problems [49]. LENA is a recording device that carries an associated privacy risk. Second, parenting self-efficacy is commonly considered in parenting intervention programs [50]. Parenting self-efficacy refers to parents’ assessment of their ability or effectiveness to successfully perform the parenting role and is a social learning theory component [51]. Task-specific parenting self-efficacy refers to a single parenting domain, such as, in this instance, communication [52].

There is no published information on the acceptability of LENA for Australian families whose children are at risk of poor language outcomes. This is in comparison with the United States, where LENA is used more extensively in trials for families living in areas of disadvantage [42,53]. Understanding the factors that influence the acceptability of LENA for children with developmental risks will help improve technology uptake.

## Methods

### Recruitment

Participants were identified as mothers with a child aged between 1 and 30 months, with at least 1 risk factor for poor child developmental outcomes. These included a history of or current mental health disorders or problems, major stressors in the perinatal period, substance misuse, government benefit, and education less than Year 12 level (Table 1).

**Table 1:** Risk factor distribution (n=8).

Number of risk factors^a^	Participants, n (%)
1	2 (25)
2	1 (13)
3	3 (38)
4	2 (25)

^a^Risk factors defined as current or history of maternal mental health problems, perinatal major stressors, substance misuse, maternal education less than Year 12, and low household income.

Participants were recruited purposively using maximal variation sampling to ensure a mix of socioeconomic and ethnodemographic backgrounds. See *Participant Characteristics* below that further describes this broad distribution. Mothers were recruited widely from those attending a variety of child and family health services for vulnerable families. These included parenting groups run by drug and alcohol services, child and family nursing home visiting programs, and community pediatrics clinics for children at risk. A participant information sheet describing the study was given to the coordinator of each of these services, outlining the study and eligibility criteria. All mothers were invited through a third person, such as the coordinator of a playgroup, to prevent coercion. All mothers were provided with a participant information sheet that included the requirements and purpose of the study. Participation was voluntary. There was no relationship between the researcher and recruited mothers.

Each participant was asked to make 2 full-day recordings (16 hours each) and to keep a record of the day’s activities to support feedback. After each recording, the mothers were provided with an individual LENA linguistic feedback report. The researcher assisted the mothers’ understanding of the results and supported goal setting for the second recording. LENA feedback reports included graphs of hourly word counts, including adult words, child vocalizations, conversational turns, and representation of the audio environment. A summary of the total daily word counts comparing the first and second recordings was also provided after the second recording. Percentile ranks were not provided, as Australian norms were not available. The discussion of the results formed the basis of the postrecording interviews. A total of 8 mothers were recruited, of which 6 were able to complete 2 recordings. One mother withdrew from the study because of relapse of a mental health condition. Another mother was unable to complete the recording as the clothing was not large enough for her child.

### Participant Characteristics

The 8 participating mothers were aged between 24 and 40 years (mean age 33.4 years). Of these 8 mothers, 2 (25%) were of non–English-speaking backgrounds, 1 (13%) had an Aboriginal background, 5 (63%) reported having either nil income or received government benefits, 2 (25%) reported a household income between Aus $1000 (US $734.50) and Aus $2000 (US $1469) per week, and 1 (13%) reported a household income of greater than Aus $2000 (US $1469) per week. Furthermore, 50% (4/8) of the mothers had an education level less than Year 12, 38% (3/8) completed high school or a diploma, 13% (1/8) had a bachelor’s degree, and 75% (6/8) of mothers reported a history of or current mental health problems. The number of risk factors for each mother is documented in Table 1. Participating children’s ages were relatively evenly distributed in 6-month bands from 0 to 30 months (mean age 14 months). There was an even distribution of child gender. Of the 8 children, 5 (63%) lived with 2 adult parents at home. One child also had grandparents involved in their care, but they did not live in the same household.

### Data Collection

A basic qualitative interpretive design was chosen to understand the mothers’ perceptions of LENA. In-depth interviews were chosen to explore individual perceptions of the use of the device. The researcher sought to understand how mothers interpret their experience of using LENA and understand the characteristics and patterns of mothers’ use of LENA. In particular, we gathered insights from mothers with identified risk factors for developmental vulnerability and poor child language outcomes. Interviews were semistructured and conducted face to face before, during, and after the use of LENA to assess acceptability from different temporal perspectives. An interview guide was also used. The interview guide covers three main areas. These were mothers’ impressions of LENA, difficulties encountered with recording, and likelihood of using LENA in the long term. In-depth interviews were conducted by the researcher at the location of the participant’s choice. Often, this was at the play group or community health center; sometimes, it was in their home. Each in-depth interview lasted between half an hour and 1 hour, and each mother was interviewed on 4 separate occasions: before the first recording, after the first recording, before the second recording, and after the second recording. As each participant was interviewed multiple times, data saturation for each participant was achieved. Where English was not the participant’s main language, interviews were conducted with the help of an interpreter. Field notes were also recorded.

Audio-recorded interviews were transcribed unless the participant disagreed with the recording. One mother disagreed with the interview recordings. Notes were instead handwritten during these interviews. Participants were allocated codes and names deidentified in the transcript to maintain their anonymity. Observational notes were also recorded in a journal.

### Data Analysis

#### Overview

Data were analyzed using thematic analysis and constant comparison. Transcripts were manually coded using an inductive approach. Each transcript was analyzed individually to identify the thematic statements. Data were analyzed by the first author and reviewed by the second author. As themes emerged, these were checked against the original transcripts and previous codes. Emerging themes were explored by the participants during sequential interviews. Successive readings led to the emergence of more focused themes. Thematic statements were clustered and checked against the original transcripts. Each cluster was analyzed to determine its essential meaning. From these clusters, major themes were generated that incorporated themes for all participants. Field notes contained a critical examination of ideas that emerged as the research progressed and the researcher’s reflections and insights. A parallel literature review of technology acceptance identified extended models of UTAUT as helpful to understanding factors influencing the likelihood of technology use. Themes generated inductively were mapped to an extended UTAUT model using an abductive approach.

#### Rigor

Transcripts and their recordings were reviewed to ensure the accuracy of the written text used in the analysis. To examine internal coherence, emergent themes were compared for consistency with the data. Two researchers coded portions of the same transcript to establish intercoder reliability. The differences and similarities in codes were then discussed. Themes were discussed by the first author and second author and reviewed with an expert mentor. An audit trail was maintained, which consisted of a diary to record the researcher’s theoretical choices, decision-making, and conclusions drawn.

#### Reflexivity

The researcher has a medical background with an interest in child development. LENA appears to be a helpful intervention for stimulating child-directed language in the home environment. LENA has been used in populations in the United States, with children who are at risk for poor developmental outcomes with positive short-term outcomes. No such published research has been conducted in Australia. The researcher is interested in identifying the conditions under which mothers with children who are at risk of poor developmental outcomes are likely to adopt LENA. This will help in identifying future caregivers who are willing to draw on the capacities of LENA to improve caregiver language output and enhance their child’s language environment.

## Results

### Overview

**Figure 1:**
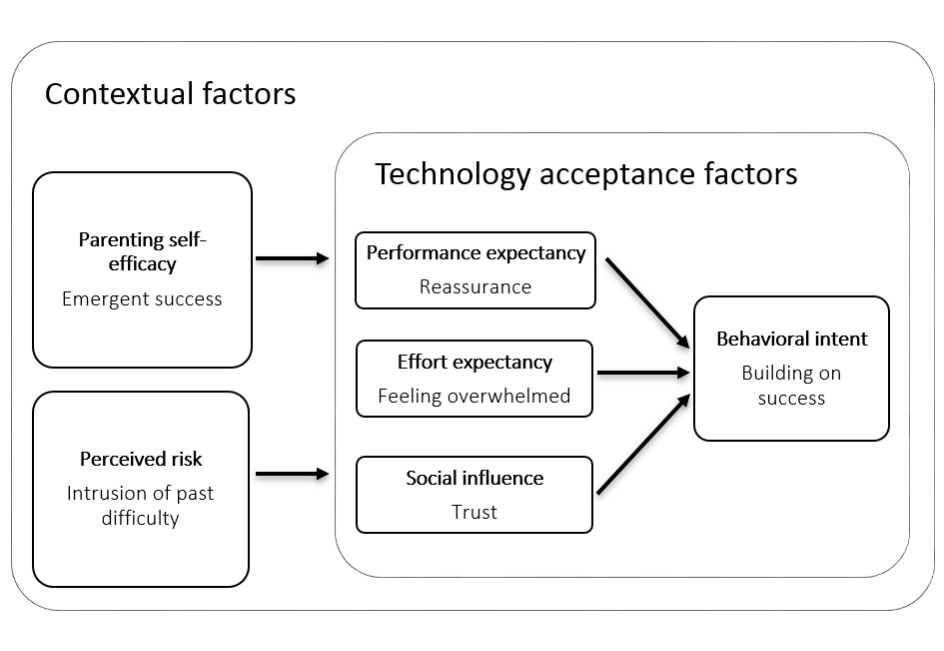
An extended unified theory of acceptance and use of technology model for Language Environment Analysis acceptability.

Thematic analysis revealed technology-related and context-related themes that impact mothers’ acceptance of LENA. Figure 1 shows these themes within an extended UTAUT model. Within technology acceptance factors, themes of reassurance, feeling overwhelmed, and trust mapped to performance expectancy, effort expectancy, and social influence. Within contextual factors, themes of emergent success and intrusion of past difficulty were mapped to parenting self-efficacy and perceived risk. The theme of building on success best described the behavioral intention or acceptability of LENA.

### Contextual Factor Themes

#### Emergent Success and Parenting Self-Efficacy

The theme of emergent success was mapped to parenting self-efficacy. Parenting self-efficacy describes a parent’s assessment of their ability or effectiveness in successfully performing the parenting role. Those mothers who demonstrated emerging confidence in their parenting were interested in exploring how LENA could improve their communication with their child. They had a sense of positivity and demonstrated building upon previous success:

I think when you are feeling good about yourself, you, you manage to sort of come from rock bottom. You are not as vulnerable. Basically, you’ve got your confidence back, you feel confident that you know you’ve already done some good things in getting, you know, communication with the child back and you are building upon that. You are feeling positive and you’ve got that momentum going forward in the direction that you want. You’ve turned it around and now you feel like, “Okay well now I’m doing so well, I can, let’s capitalise on this and do it [LENA].”

P5

Mothers with a background of mental health issues, particularly anxiety and depression, experienced periods of low parenting self-efficacy. Being recorded was associated with the impression of being judged. Some mothers expressed anticipatory concern that feedback could be critical and reveal flaws in parent-child interactions:

Because in my headspace I thought I was doing everything wrong and I [would have] thought, “Oh, this is just another way for someone to find out what I’m doing wrong.”

P5

One mother expressed confidence in her parenting self-efficacy based on previous experience. She felt that she was able to assess the development of her child better without the need for technology:

My child’s condition, I can see it. He is making his own progress. I don’t know if it [LENA] helps. I have two other children and from previous experience I never needed it [LENA]...I don’t need the information to monitor his progress.

P3

#### Intrusion of Past Difficulty and Perceived Risk

The theme of intrusion of past difficulty was mapped to perceived risk. Perceived risk with technology commonly includes concerns about privacy problems [49]. Past difficulties impacted mothers’ experience of using LENA. Some mothers described issues involving child protection agencies, incarceration, mental health difficulties, and drug rehabilitation. There was a perceived risk associated with the recording.

For 2 mothers, the recording device carried a stigma of being spied on, and concerns were expressed about the loss of privacy and tampering with recorded information. The recording device was sometimes considered suspicious by others. Concerns relating to child protection and domestic violence were also raised:

But – “Oh, watch out! She’s wired!” – muck around...I tried to point it [LENA] out...but none of them would listen, so I just thought pfft. If you’s are so weird about me recording her or her recording me and her, and then yeah. And they didn’t answer, so I just walked out.

P2

### Technology Acceptance Factors

#### Reassurance and Performance Expectancy

The theme of reassurance was mapped to performance expectancy. Performance expectancy describes the perceived usefulness of technology [47]. All mothers, except one, expressed anxiety about their communication ability. Commonly, these mothers were interested in receiving feedback on their interaction with their child and sought reassurance about the efficacy of their communication with their child:

Um, I’m just hoping to see how much I can improve to help [child’s name], like I’d like to know, cause I can’t really, sounds silly, but I can’t really hear myself, I don’t know. You know, I want to know how much I’m putting into making him speak, so whatever I could improve to help [child’s name], that’s what I want to do.

P7

Was I talking enough?

P2

Mothers were often concerned about their child’s development and perceived their child to be at risk of poor language development. This could be in the context of the child’s pre-existing medical problems, such as neonatal withdrawal, behavioral difficulties in the child, or a family history of developmental issues such as language delay. These mothers felt that LENA was useful in improving their child’s communication. However, for 1 family, the experience of LENA represented a deficit model, which was incompatible with their strong focus on positive health and well-being. The LENA system was seen to focus on potential issues:

I don’t want to predict [language difficulties]. I don’t want that trouble...My husband asked me, ‘Why worry? His problems have all gone now and the doctor said he will be normal.’ He does not think he needs the recording, he is not concerned and believes he will pick up [language] as he grows up.

P3

#### Feeling Overwhelmed and Effort Expectancy

The theme of feeling overwhelmed was mapped to effort expectancy. Effort expectancy describes the ease of use of technology [47]. Not all mothers were in a position to be able to take on the additional demands required to participate. For example, 1 mother experienced a relapse and withdrew from the study. Others expressed interest but declined because of other commitments. Rescheduling appointments was common where recordings were not completed before the next review.

On the recording day, some mothers found it difficult to balance competing demands. These demands were not always avoidable or predictable. These included teething, illness, and unsettled behavior in children. Sometimes, the needs of a sibling led to reduced interaction with the child being recorded: “We weren’t having a good day, he was in such a state and I know, there’s no point putting it [LENA] on” [P1].

#### Trust and Social Influence

The theme of trust was mapped to social influence. Social influence is the “degree to which an individual perceives that important others believe he or she should use the new system” [48]. Mothers experienced various difficulties, including mental health issues, addiction, child medical health problems, and relational breakdown. Some mothers reflected on parenting issues such as being unavailable to their children both physically and emotionally, uncertainty in how to parent, and not identifying medical and developmental issues early enough.

Despite these difficulties, several mothers described a continuing process of coming out of these difficulties, often through the help of a caseworker or health professional. Building upon a trusted professional relationship was important to 4 of the mothers. The caseworker or trusted professional was seen as a bridge to the introduction of LENA:

Because I know that umm [caseworker’s] interested in [child’s] progress and my progress, umm and would only recommend something that was beneficial to both of us. Umm whereas if anyone else sort of, you’d be like, “Oh.” And because she knew by being in the home...what was going on specifically, so she thought that potentially that [LENA] could be umm helpful, but yeah with someone else, you’d just be like, “Oh okay.”

P5

Participants were more likely to trust and use the technology when they were positively influenced by a trusted professional:

If it wasn’t safe my caseworker wouldn’t let me do it, that’s why I give everything to her, run it by her first...she knows what’s happening and if it’s good for me and baby.

P2

#### Building on Success and Behavioral Intention

The theme of building on success was mapped to behavioral intention. Behavioral intention has been defined as “the degree to which a person has formulated conscious plans regarding whether to perform a specified future behavior” [49]. Positive results were comforting for some mothers who regarded their results with pride and satisfaction. Several mothers valued results as a validation of their self-efficacy:

I like the fact that I know that she’s communicating back and I’m communicating to her and it just reassures me that I’m doing the right thing, yeah, so it’s good to know...It makes me feel good and yeah, happy that I’m doing something right.

P4

Success in achieving a good word count motivated some mothers to do more with their children: “So, seeing the results would probably make me want to do it more and more to see if I can get it up to that level” [P2]. However, not all parents found the goal of a word count as motivational:

See, I’m still bogged down in all of that sort of stuff rather than, “Oh yay, I haven’t got my word count.” I couldn’t care less if I got the ** word count in.

P5

## Discussion

### Principal Findings

This is the first Australian study to describe the factors that influence the acceptance of LENA for mothers with children at risk of experiencing delayed language development. The results of this study are relevant to understanding the acceptability of the LENA system for developmentally at-risk children in Australia. The findings may be used to develop interventions and foster conditions that aim to improve early language development. The findings of this study can help increase the likelihood that mothers with at-risk children will use LENA to improve their language output and enhance their children’s language environment.

The UTAUT model is a well-established, statistically validated model that describes the conditions that influence behavioral intention and use of technology [48]. This study developed an extended UTAUT model to represent technology-specific and context-specific factors that influence the acceptance of LENA by mothers with children at risk of poor developmental outcomes. Contextual factors were as important as specific technology factors for acceptance of LENA. A mother’s experience of using LENA was highly influenced by her individual circumstances. These contextual factors were represented by themes of emerging success and the intrusion of past difficulty. Emerging success was considered to be encompassed by the condition of parenting self-efficacy. Parenting self-efficacy was interwoven with the acceptability of LENA and influenced the appraisal of the technology. Mothers with emerging success found satisfaction with their results. These mothers had some anxiety about their communication self-efficacy and expected LENA to help them improve their communication with their children. However, very low or high parenting self-efficacy appeared to negatively influence the performance expectation of the technology and reduced the likelihood of LENA acceptance. For one mother, the condition of low parenting self-efficacy led to the impression that LENA was an additional burden. High parenting self-efficacy was also negatively correlated with acceptability. For 1 mother who felt confident in her parenting ability, LENA was not expected to provide any beneficial new information, and she expressed low interest in ongoing use.

This study also revealed other contextual factors that hindered acceptability. The intrusion of past difficulty was mapped to perceived risk. These were seen as negative past experiences for mothers, which led to the perception that LENA was being used to monitor the mother rather than help the child. This is consistent with the research by Allen et al [54], that identified issues of intrusion and recording privacy.

Technology acceptance factors helped explain the conditions that influence the acceptability of LENA. These factors include perceived usefulness, ease of use, and social influence. The theme of reassurance was mapped to performance expectancy. Mothers showed increased interest in LENA when they perceived that the technology provided useful feedback on their communication. However, some aspects of LENA results were not reassuring and may sometimes place a parent at risk of exposing their child’s language difficulties.

The theme of feeling overwhelmed described effort expectancy. Overall, mothers described LENA as easy to use and clothing as appealing. They felt that the recording process was natural, and most mothers forgot that they were being recorded. However, contextual difficulties negatively influence the effort expectancy. Increased demands, often in the context of high levels of stress, impacted usability with mothers waiting for ideal recording conditions.

Social influence, as represented by the theme of trust, is an important acceptance factor. Building on a trusted professional relationship improved the acceptance of LENA. This is in agreement with a UK study looking at the acceptability of LENA by parents of young deaf children, which reported the importance of establishing trust through a known professional [54].

Experiencing cumulative success improves the acceptability of LENA. LENA provides short-term goals, such as word count goals, and assists mothers’ motivation and confidence as they experience small successes. This may be especially important for mothers to improve their communication self-efficacy. Feedback received from LENA can support parents to persist in improving their child’s language environment. Bandura described the powerful influence of the ability to master a task [51]. Mothers who saw evidence of reciprocal conversation felt motivated to increase this. Satisfaction with reports and experiencing cumulative success improves the acceptability of LENA. Conversely, there is a risk of repeated nonmastery demotivating parents and becoming another way to demonstrate what they are *doing wrong*.

LENA was positively perceived by most mothers, with 83% (5/6) of mothers who were able to complete a trial of LENA expressing an interest in continued use. Mothers felt that LENA was beneficial for their children and found feedback useful in increasing awareness of the home language environment, improving confidence in their interactions with their child, and providing reassurance about their child’s language development.

Although mothers valued feedback, the researcher observed the need to interpret the results. Presentation of graphical information in bar charts with word counts throughout the day was not always readily accessible to participants. It failed to answer the question, *Was I talking enough?* This finding is consistent with observations by Allen et al [54], which emphasized the need for clinician interpretation of results. Other pilot studies have incorporated individual feedback provided by research assistants to help caregivers understand their specific results [37,55]. Clinicians need to add value to the interpretation of results.

### Limitations

Emphasis in this study was placed on understanding the experience of an individual mother’s use of LENA with children at risk of poor developmental outcomes in central and inner west Sydney. The study was able to sample from a wide ethnodemographic population, as the software can be used with linguistically diverse families. The results are informative but not necessarily generalizable because of the small sample size.

The nature of the research was explorative, and the trial duration was not long enough to assess any improvements in child and adult language measures. These results are informative for the development of future interventions. These should include regular outcome measures, including pre-, post-, and follow-up intervention measures for children and adults to demonstrate changes over time.

A full-day recording was chosen to reduce the *Hawthorne effect*, where individuals may modify their behavior in response to knowingly being observed. However, for some mothers, this likely contributed to mothers’ feeling overwhelmed by requiring recordings to be completed in a specific 16-hour period.

Fathers in 2-parent households were underrepresented in this study. Their voices were expressed through their partners, but they were not directly interviewed. Assessment of their perspective is important, as their attitudes toward LENA also influence the recording and acceptability of language intervention. Allen et al [54] identified the importance of the involvement of both parents, particularly in the beginning, to establish understanding and consent.

### Implications for Future Research

LENA is a linguistic technology that provides parents and associated professionals with information about a child’s home language environment. There are now published Australian norms of daily adult word count and child vocalizations [56]. The growth of expressive communication in the first 3.5 years is very similar to that in American children [57]. The positive correlation identified in American research between adult word count and child vocalization is presumed to be held in the Australian context and is the basis of quantitative feedback. On an individual level, absolute word counts can be used to track progress. There is a need for a simplified reporting system so that parents can determine whether they are on target or not. Having normed Australian data integrated into the LENA system to show an individual level may help parents to better assess their progress.

Further research is needed to determine how community-based interventions can incorporate the use of LENA. Future research needs to measure pre- and postintervention language scores to assess the effectiveness of LENA as a strategy for reversing developmental risk in populations at risk for poor child developmental outcomes. Trials will benefit from incorporating learning from this study, including strategies for supporting parents. This includes partnering with parents through a trusting clinician relationship and helping mothers build on their parenting self-efficacy. Gauging mothers’ acceptability of LENA through trial use will help establish which mothers are both ready and interested in using LENA long term. LENA is best placed as an adjunct to a parent-delivered language program and has been described as a strategy to increase the effectiveness of behavioral interventions [37].

### Conclusions

An extended UTAUT model was created specifically for the use of LENA for children at risk of poor language development. This model illustrates the underlying factors contributing to the acceptability of LENA, including technology-specific and contextual factors. Key findings included an increased likelihood of taking up LENA when (1) LENA is introduced by a trusted clinician, (2) LENA results are perceived as useful and motivating to mothers, (3) mothers feel confident in achieving communication goals, and (4) clinicians need to be mindful of the perceived risk associated with recording technology. Language enrichment interventions incorporating LENA may increase parenting self-efficacy, and partnering with families will likely improve technology acceptance and intervention success.

## Abbreviations

LENA: Language Environment Analysis
SES: socioeconomic status
UTAUT: unified theory of acceptance and use of technology
